# Disparities in HIV Care: A Rural–Urban Analysis of Healthcare Access and Treatment Adherence in Georgia

**DOI:** 10.3390/healthcare13121374

**Published:** 2025-06-09

**Authors:** Donrie J. Purcell, Maisha Standifer, Evan Martin, Monica Rivera, Jammie Hopkins

**Affiliations:** 1 Satcher Health Leadership Institute, Morehouse School of Medicine, Atlanta, GA 30310, USA; mstandifer@msm.edu (M.S.); emartin@msm.edu (E.M.); jhopkins@msm.edu (J.H.); 2 Rollins School of Public Health, Emory University, Atlanta, GA 30322, USA; mrivera@emory.edu

**Keywords:** HIV disparities, rural–urban healthcare access, stigma, treatment adherence, health equity, Georgia USA

## Abstract

**Background**: This study examines disparities in HIV-related healthcare access, support, and treatment adherence between rural and urban Black/African American populations in Georgia, USA, emphasizing structural, socioeconomic, and stigma-related barriers affecting health outcomes. **Methods**: A cross-sectional quantitative study was conducted using structured surveys administered via RedCap from August to December 2023. Participants (*n* = 55) were recruited through community-based HIV organizations, healthcare providers, and advocacy networks. The survey assessed demographic factors, healthcare access, adherence to treatment, support systems, and experiences with stigma. Data were analyzed using IBM SPSS Statistics, version 28.0 (IBM Corp., Armonk, NY, USA), with chi-square tests examining associations between geographic location and HIV-related outcomes. **Results**: Findings indicate significant disparities in HIV care access and support. Urban participants were more likely to receive family and friend support (*p* < 0.01), financial assistance through the Ryan White Program (*p* = 0.01), and timely linkage to care within one week of diagnosis (*p* < 0.05). Rural participants reported lower educational attainment, income levels, and limited healthcare access, contributing to poorer health outcomes. No significant differences were observed in PrEP or condom use. **Conclusions**: The study underscores the need for targeted interventions. Expanding telehealth, enhancing community outreach, and reducing stigma through policy reforms are critical to improving HIV-related health outcomes in rural Georgia.

## 1. Introduction

HIV continues to pose a major public health challenge in the United States. In 2022, the Centers for Disease Control and Prevention (CDC) reported approximately 36,136 new HIV diagnoses in the United States, with over 50% occurring in the Southern U.S., a trend that has persisted for over a decade [1]. Georgia alone reported a rate of 22.1 per 100,000, compared to the national average of 11.5 per 100,000. This marks only a slight decline from 2012 [2,3], indicating persistent disparities despite advances in treatment and prevention. Over half of all new HIV diagnoses occur in this region, driven by socioeconomic inequalities, healthcare shortages, and cultural stigmas that hinder access to timely care [4]. States like Georgia and Louisiana experience particularly high infection rates due to entrenched poverty, limited healthcare infrastructure, and persistent HIV-related stigma [5].

Georgia ranks among the top states for new HIV diagnoses, especially affecting Black men who have sex with men (MSM), a group disproportionately impacted by systemic barriers such as economic instability, limited healthcare access, and stigma [6]. In 2022, Black MSM accounted for approximately 44% of all new HIV diagnoses in Georgia in 2022, underscoring the severe disparities experienced by this group [7]. Public health institutions, including the Satcher Health Leadership Institute (SHLI) at Morehouse School of Medicine is a national leader in advancing health equity for vulnerable populations through research, training, and policy development. The SHLI collaborate with local and national organizations to promote health equity through policy reform, stigma reduction campaigns, and expanded healthcare access initiatives aimed at addressing these disparities [8].

Urban areas like Atlanta benefit from stronger healthcare infrastructure, offering specialized HIV services such as antiretroviral therapy (ART), pre-exposure prophylaxis (PrEP), and community-based support programs [9]. However, barriers such as stigma, housing insecurity, and socioeconomic disparities persist, limiting the effectiveness of available resources. A cornerstone of these services is the Ryan White HIV/AIDS Program. It is a federal initiative that provides a comprehensive system of care for low-income people with HIV who are uninsured or underinsured. The program primarily targets individuals who do not have sufficient healthcare coverage or financial resources for HIV treatment [10]. Rural communities face even more significant challenges, including fewer healthcare facilities, limited specialized providers, and long travel distances to access care [11]. Cultural stigma and social isolation further exacerbate these difficulties, discouraging individuals from seeking HIV-related services. Structural inequities such as poverty and unemployment also limit individuals’ ability to afford care and maintain consistent treatment. In Georgia, Black/African American individuals accounted for 65.2% of all new HIV diagnoses in 2023, despite representing only 32.6% of the state’s population [12]. This striking disparity highlights the urgent need to tailor interventions to the unique barriers faced by this community.

This study was conducted as part of an Ending the HIV Epidemic (EHE) initiative and specifically focuses on Black/African American populations in Georgia, a disproportionately affected group. We examine disparities in HIV-related healthcare access, support systems, and treatment adherence between rural and urban communities, with emphasis on structural, socioeconomic, and stigma-related barriers. To bridge these gaps, innovative models like telehealth, mobile clinics, and culturally appropriate community-led initiatives have shown promise in improving care access and reducing rural–urban disparities [13]. Expanding these services while addressing social determinants of health is essential to ensuring health equity and reducing HIV transmission across diverse communities in Georgia.

## 2. Materials and Methods

### 2.1. Study Design

This study employed a quantitative cross-sectional design to examine disparities in HIV-related health outcomes between rural and urban populations in Georgia. The study was approved by the Morehouse School of Medicine Institutional Review Board (FWA #4535, IRB I.D. #674), and all participants provided written informed consent before participation. Data were collected between August and December 2023 using a structured survey administered through the RedCap platform. The study explored socio-demographic characteristics, healthcare access, and treatment adherence to identify potential contributors to observed disparities.

### 2.2. Sample and Recruitment

Participants were recruited through community-based HIV organizations, healthcare providers, and advocacy networks across Georgia. A purposive sampling approach was used to identify and partner with organizations that primarily serve Black/African American individuals and are actively engaged in Ending the HIV Epidemic (EHE) initiatives. Within those settings, participants were recruited using convenience sampling, based on their availability and willingness to participate.

Eligibility criteria included individuals aged 18 years or older, who had a confirmed diagnosis of HIV, and resided in either a rural or urban area of Georgia. Recruitment efforts aimed to ensure diverse representation across key demographic and geographic subgroups relevant to the study’s focus on healthcare access and HIV-related outcomes.

This study was supported by targeted funding from the Gilead COMPASS Initiative^®^, which aims to reduce HIV-related disparities in Black communities across the U.S. South. In alignment with this mission, partnerships were formed with trusted community-based organizations that primarily serve Black/African American individuals in both rural and urban counties in Georgia. To help mitigate potential recruitment bias, efforts were made to ensure inclusion across geographic areas, participant roles (e.g., providers, clients, advocates), and diverse experiences with HIV care and stigma.

### 2.3. Survey Instrument

Data were collected using the Ending the HIV Epidemic (EHE) Interview Guide Survey, a structured tool designed to assess key factors influencing HIV care engagement. The survey was developed by the research team at the Satcher Health Leadership Institute in collaboration with Emory University consultants and local community partners engaged in Ending the HIV Epidemic efforts across Georgia. The design was informed by both national EHE priorities and region-specific input obtained through early stakeholder engagement. The survey covered various domains, including demographics, healthcare access, treatment adherence, community support, experiences with discrimination, and mental health and substance use. Participants completed the survey in approximately 60–90 min, providing insights into the social and structural determinants affecting their HIV care. For this study, the following sections were analyzed:

- Demographics: Age, gender identity, race/ethnicity, educational background, and socio-economic status.
- Access to Care: Availability and barriers related to HIV testing and treatment.
- Adherence to Treatment: Medication access, adherence challenges, and support systems.
- Community Support: Sources of family, friend, and healthcare provider support post-diagnosis.
- Experiences with Discrimination: Stigma encountered when seeking HIV care.
- Mental Health and Substance Use: Use of mental health services and how substance use impacted HIV care.

### 2.4. Data Analysis

Survey responses were compiled and analyzed using SPSS statistical software. Descriptive statistics were computed to summarize socio-demographic characteristics and healthcare access indicators. Chi-square tests of independence were conducted to evaluate relationships between socio-demographic variables (e.g., geographic location, race/ethnicity, gender identity) and HIV-related outcomes such as treatment adherence, care access, and experiences with stigma. Significance thresholds were set at *p* < 0.05, and results were interpreted with a focus on identifying key disparities and informing public health interventions. Findings are presented in the results section, with recommendations for further research to strengthen the evidence base on HIV care disparities in Georgia.

## 3. Results

### 3.1. Participant Demographics

A total of 55 participants were included in the study, with 20 residing in rural areas and 35 in urban areas of Georgia. Table 1 provides a summary of the demographic characteristics of the study population. The mean age of participants was 42.5 years (SD = 8.3), with rural participants being older on average (46 ± 9.5 years) compared to their urban counterparts (39 ± 7.2 years). The sample was predominantly male (60%), with rural areas reporting a higher proportion of males (70%) compared to urban areas (55%). Black/African Americans constituted 95% of the total sample across both rural and urban settings, while 5% identified as mixed or other racial backgrounds. Urban participants were more likely to report higher educational attainment, with 60% having completed college or higher, compared to 35% in rural areas. Additionally, 50% of urban participants reported annual incomes above $50,000, compared to only 25% of rural participants.

### 3.2. Diagnosis Support

Urban participants reported significantly greater support from family (χ^2^ = 6.898, *p* = 0.009) and friends (χ^2^ = 6.158, *p* = 0.013) compared to rural participants. Alternative sources of support were also more prevalent among urban individuals (χ^2^ = 9.448, *p* = 0.002). No significant difference was observed in support from co-workers (χ^2^ = 1.721, *p* = 0.190).

### 3.3. Financial Assistance

Urban participants were significantly more likely to utilize the Ryan White Program for financial assistance (χ^2^ = 6.559, *p* = 0.010). No significant differences were found in financial burdens related to food (χ^2^ = 1.683, *p* = 0.194), travel (χ^2^ = 1.148, *p* = 0.284), or prescription costs (χ^2^ = 3.002, *p* = 0.083). Private insurance and state support program utilization did not differ significantly between rural and urban groups (*p* = 0.780 and *p* = 0.402, respectively).

### 3.4. Healthcare Access

Significant disparities were observed in healthcare access. Urban participants were more likely to be linked to care within one week of diagnosis (χ^2^ = 10.587, *p* = 0.014). However, there was no significant difference in transportation methods between rural and urban participants, with urban residents reporting slightly higher use of public transportation (*p* = 0.482).

### 3.5. HIV Prevention Strategies

Urban participants were more likely to engage in avoidance strategies to prevent HIV transmission (χ^2^ = 3.869, *p* = 0.049). However, no significant differences were observed in condom use (*p* = 0.822), education-based prevention (*p* = 0.221), or the use of pre-exposure prophylaxis (PrEP; χ^2^ = 0.104, *p* = 0.747).

### 3.6. Community Services

Urban residents were significantly more likely to use counseling services (χ^2^ = 9.448, *p* = 0.002) and medical management programs (χ^2^ = 4.589, *p* = 0.032). Mental health service utilization did not differ significantly between groups (*p* = 0.528). Housing support services were also evenly distributed (*p* = 0.391).

## 4. Discussion

The findings of this study highlight critical disparities in HIV-related support systems, financial assistance, and healthcare access between rural and urban populations in Georgia. These results align with existing literature emphasizing the persistent inequities faced by individuals in rural areas compared to their urban counterparts. Urban participants demonstrated significantly greater family and friend support, faster linkage to care, and higher utilization of financial assistance programs like the Ryan White Program [10]. These findings mirror trends identified in national studies, where urban areas benefit from greater healthcare infrastructure and social support systems. For example, the Health Resources and Services Administration [14] reported that only 8.2% of Ryan White HIV/AIDS Program providers operate in rural areas, despite these regions comprising a substantial portion of the population. This scarcity of providers can result in delays in diagnosis and treatment, adversely affecting health outcomes.

Neighborhood characteristics, such as socioeconomic status, racial segregation, and healthcare access, are significant determinants of HIV-related health outcomes [15]. Structural inequalities, such as poverty, housing instability, and transportation barriers, compound the effects of HIV in rural settings [16]. These factors were reflected in this study, where rural participants reported lower engagement with community services and slower linkage to care. Similarly, studies indicate that economically disadvantaged regions with fewer healthcare resources experience poorer HIV care outcomes due to delayed diagnoses and inconsistent treatment access [15]. Existing literature further supports these patterns, for example Reif et al. [6] found that Southern rural communities experience higher HIV burden and worse care engagement compared to urban areas, largely due to infrastructure deficits and socio-environmental challenges. Unlike previous studies, our findings contribute context-specific insights into how stigma, community support systems, and access to financial assistance shape HIV care disparities among Black/African American populations in Georgia’s rural and urban communities. These findings point to the need for coordinated policy reforms aimed at improving health system infrastructure and reducing place-based inequities.

The role of stigma in rural areas cannot be overstated. The close-knit nature of rural communities often heightens the fear of being recognized when seeking HIV-related services, discouraging individuals from accessing care [17]. Social discrimination and fear of disclosure often deter individuals from seeking care, compounding delays in diagnosis and treatment. Turan et al. [18] highlight that HIV-related stigma not only hampers adherence to treatment but also negatively influences health outcomes. Owens et al. [19] report that rural adolescent sexual minority males face higher rates of condomless sex and lower utilization of HIV prevention services compared to their urban counterparts. This supports findings that healthcare-related discrimination, driven by social and cultural stigmas, continues to hinder care access in rural communities [15]. Additionally, the Rural Health Information Hub [20] emphasizes that stigma, combined with limited healthcare infrastructure and socioeconomic constraints, paired with lack of anonymity and limited confidentiality in rural health settings, significantly worsens disparities in HIV care. Additionally, misinformation and lack of education about HIV transmission contribute to persistent stigma, making it harder for people to access care without judgment [21]. These findings underscore the need for tailored interventions addressing the unique social determinants of rural communities.

The disparities observed highlight the urgent need for public health interventions that are both targeted and scalable. Sangaramoorthy et al. [22] highlight that rural residents often face more severe stigma, longer travel times, and fewer specialized providers than urban residents, leading to reduced ART adherence and care continuity. Similarly, Ohl and Perencevich [23] found that people with HIV in rural areas are significantly less likely to receive guideline-concordant care and timely antiretroviral therapy compared to urban populations. Based on our findings, we recommend expanding funding for healthcare infrastructure in rural areas, increasing education about available services like the Ryan White Program, and implementing stigma-reduction campaigns are critical steps. Telehealth solutions, which have shown promise in bringing services directly to rural communities, could also play a significant role in bridging the gap in access to care. Structural interventions aimed at addressing economic and social challenges at the neighborhood level may also improve outcomes [16]. Furthermore, findings from Zang et al. [24] suggest that addressing geographic inequities in access to resources and care may reduce disparities in both rural and urban settings.

### Limitations and Future Research

While the study provides valuable insights, it is not without limitations. The sample size, though sufficient for chi-square analysis, may not capture the full heterogeneity of rural and urban populations. A purposive sampling strategy was used to identify and collaborate with community-based organizations serving Black/African American communities, and convenience sampling was employed to recruit participants from within those organizations. While appropriate for engaging a high-priority group, this approach may introduce selection bias and limits generalizability.

The study focused on Black/African American individuals, consistent with the goals of the Ending the HIV Epidemic (EHE) initiative. While this strengthens the study’s relevance for disproportionately affected populations, the findings may not extend to other demographic groups. Additionally, self-reported data on social support, stigma, and financial assistance may be subject to recall bias.

Future research should explore longitudinal designs to assess the long-term impact of rural–urban disparities and examine the effectiveness of targeted interventions designed to improve care access across diverse populations.

## 5. Conclusions

This study highlights the multifaceted challenges faced by individuals with HIV in rural areas of Georgia compared to their urban counterparts, particularly within Black/African American communities disproportionately affected by the epidemic. Addressing these disparities requires a holistic approach that considers social, economic, and structural determinants of health. Policymakers, public health agencies, and community stakeholders must prioritize equitable resource distribution and stigma reduction strategies to ensure that no community is left behind in the fight against HIV. Sustained investment in tailored interventions, particularly in underserved rural areas, will be critical to advancing the goals of the Ending the HIV Epidemic (EHE) initiative.

## Figures and Tables

**Table 1:** Socio-demographic characteristics and Chi-Square analysis of HIV care access by geographic location (Rural vs. Urban).

Demographic Variable	Rural (*n* = 20)	Urban (*n* = 35)	Overall (*n* = 55)
**Age (Mean ± SD)**	46 ± 9.5	39 ± 7.2	42.5 ± 8.3
**Gender (Male %)**	70.0	55.0	60.0
**Gender (Female %)**	30.0	45.0	40.0
**Black/African American (%)**	95.0	95.0	95.0
**Mixed/Other (%)**	5.0	5.0	5.0
**High School Education or Less (%)**	65.0	40.0	50.9
**College Education or Higher (%)**	35.0	60.0	49.1
**Income > $50,000 (%)**	25.0	50.0	40.0

**Note**. *n* = Sample size.

## Data Availability

The de-identified dataset generated and analyzed in this study was archived at the Morehouse School of Medicine and made available upon request. No custom code or computational models were used in this study.

## Abbreviations

The following abbreviations are used in this manuscript:

HIV	Human Immunodeficiency Virus.
PrEP	Pre-Exposure Prophylaxis
SPSS	Statistical Package for the Social Sciences
ART	Antiretroviral Therapy
EHE	Ending the HIV Epidemic
MSM	Men who have sex with men
SHLI	Satcher Health Leadership Institute
